# Developing and validating the Taiwan version of the meaningful activity participation assessment (T-MAPA) with Rasch analysis

**DOI:** 10.1186/s12877-023-03839-9

**Published:** 2023-03-22

**Authors:** Ya-Chin Yeh, Daniel Park, Shang-Yu Yang, Chang-Chih Kuo

**Affiliations:** 1 Department of Occupational Therapy, Shu-Zen Junior College of Medicine and Management, 452, Huanqiu Rd., Luzhu Dist, Kaohsiung, Taiwan; 2 Chan Division of Occupational Science and Occupational Therapy, University of Southern California, 1540 Alcazar Street, Los Angeles, CA 90089-9003 USA; 3 Department of Healthcare Administration, College of Medical and Health Science, Asia University, 500, Lioufeng Rd, Wufeng, Taichung, Taiwan; 4 Department of Occupational Therapy, Kaohsiung Medical University, 100, Shyh-Chung 1st Rd, Kaohsiung, Taiwan

**Keywords:** Meaningful activity participation, Healthy ageing, Rasch analysis, Cross-cultural adaptation

## Abstract

**Background:**

Meaningful activity participation has shown good predictability for healthy ageing in older adults, and their participation can be assessed using the Meaningful Activity Participation Assessment (MAPA). However, the MAPA has never been validated in any Taiwanese population. Moreover, different cultures may interpret meaningful activity participation differently. This study thus aimed to cross-culturally adapt the MAPA into a Taiwan version (i.e., the T-MAPA) and to investigate the psychometric properties of the T-MAPA in older adults in Taiwan.

**Methods:**

This study consisted of 3 phases. First, the original MAPA was cross-culturally adapted in 6 stages, including forward, synthesis of, and back translations, cognitive debriefing, expert review, and pilot testing on 18 older adults. Second, a Rasch–Andrich rating scale model was applied to evaluate the psychometric properties (including category function, unidimensionality, item functioning and targeting, and reliability) of the adapted version in a sample of 146 older adults. Lastly, the convergent validity and test–retest reliability were examined on 120 and 49 older adults, respectively.

**Results:**

After cross-cultural adaptation, the first version of the T-MAPA contained 29 items. Optimal category function was obtained by reducing the response categories of the frequency subscale to 4 and retaining a 5-point rating for the meaningfulness subscale. After the removal of 1 misfit item, a 28-item T-MAPA was generated. This version demonstrated unidimensionality, measurement invariance among different subgroups (regarding sex and education), acceptable item targeting (< 1 logit) and negligible floor and ceiling effects (1.37%; 0.68%), high reliability (person reliability coefficient = 0.86; small standard error < 0.5 with large test information > 4), confirmed convergent validity (absolute *r* = .49–0.54 with psychological well-being, depressive symptoms, and mental and physical health), and excellent test–retest reliability (intraclass correlation coefficient = 0.94).

**Conclusion:**

The cross-culturally adapted 28-item T-MAPA is suitable for application to the older population in Taiwan to measure meaningful activity participation. Future examinations of the T-MAPA in other populations with specific clinical features are warranted to extend its utility in practice.

**Supplementary Information:**

The online version contains supplementary material available at 10.1186/s12877-023-03839-9.

## Background

The worldwide increase in life expectancy and the growth of the older population has promoted a global public concern for the health and quality of life of older adults throughout the ageing process [1, 2]. In Taiwan, official statistics on population projections revealed that older adults accounted for more than 14% of the total population in 2018, when Taiwan was classified as ‘an aged society’. It is expected that older adults will account for more than 20% of the Taiwanese population by 2025, when Taiwan will become a ‘super-aged society’ [3]. These older adults may encounter age-related physical and psychological declines in function and independence, which may increase the burdens on families and the general society. However, older adults can still experience satisfactory ageing with the active management of their health and lifestyles [4]. For example, incorporating physical and mental activities in everyday life enhances life satisfaction and psychological well-being, and maintaining a healthy diet and appropriate body weight decreases cardiovascular disease in older adults [5]. To ensure the rights of health and wellness of older adults, the World Health Organization (WHO) proposed that healthy ageing should be promoted and measured scientifically [6].

The WHO states that ‘healthy ageing’ involves developing and maintaining the functional ability that enables wellbeing in older age [6–8], which can be attained by participating in occupations (i.e., chunks of activities that are cultually and personally meaningful) [9–11]. Evidence has demonstrated that maintaining a greater degree of overall meaningful activity can predict a higher level of healthy ageing, which is indicated by multiple positive outcomes such as reduced mortality; enhanced psychological function, cognitive function and social competence; and improved psychological and physical well-being and health-related quality of life [12–15].

To predict the level of healthy ageing, the assessment of participation in meaningful activities should contain items covering various domains and comprehensive concepts of activity participation. Ageing and occupation-based theories and models such as the Model of Selective Optimization with Compensation [16], the Life-Span Theory of Control [17], and the Model of Human Occupation [18] indicate that older adults may change the meaning they attach to activities over time and develop coping strategies through routine practice and the use of their capacities and resources to successfully participate in activities and maintain well-being. Accordingly, two properties of measures of meaningful activity participation for older adults are suggested: (i) a variety of activity items for personal selection, such as those belonging to the physical, psychosocial and educational domains [9], and (ii) subjective and objective indicators of meaningful activity participation (i.e., meaningfulness and frequency) [19]. In addition, to assist in intervention design and outcome measurement for older adults, a measure of activity participation should have two additional properties: (iii) sound psychometric properties and (iv) the capability to predict aspects of healthy ageing such as psychological well-being and health-related quality of life.

A review of existing measures of activity participation revealed that most do not feature the four properties described above. Some measures do not provide various activity items for selection. For example, the Engagement in Meaningful Activities Survey (EMAS) assesses aspects of activity meaning of specific pre-determined activities [20]. Therefore, the EMAS can neither address which activities are preferred by the individual nor provide information on the meaningfulness of various activities for personal selection and adaptation. Other measures do not use both subjective and objective indicators to assess concepts of activity participation. For example, the modified NPS (Norling, Pettersson, Selander) Interest Checklist [21] uses subjective measures such as preference, experience, motivation and perceived well-being on leisure activities. With only subjective measures, the modified NPS Checklist cannot provide information on how much time was spent on leisure activity participation. On the other hand, the Activity Card Sort (ACT) [22] calculates the number of engaged activity items as an objective indicator of activity participation. With only objective measures of activity numbers, the ACT cannot reflect the personal experience of meaningfulness in activity participation. To our knowledge, the Meaningful Activity Participation Assessment (MAPA) is the only measure which contains all the properties required for a comprehensive measurement of meaningful activity participation [23].

The MAPA is a checklist-type survey consisting of 28 diverse activity items. These items capture the frequency of participation and the meaningfulness of each activity experienced by the individual. The MAPA consists of two separate subscales. The frequency subscale evaluates the amount of time spent on each of the 28 activities (e.g., cultural activities and physical exercise) during the last few months on a 7-category scale from 0 (‘not at all’) to 6 (‘every day’). The meaningfulness subscale evaluates the meaningfulness of each activity on a 5-category scale from 0 (‘not at all meaningful’) to 4 (‘extremely meaningful’). These two sets of ratings are multiplied for each item and then summed to arrive at an individual’s overall perceived meaningful activity participation score. Thus, the total scores range from 0 to 672, with higher scores indicating greater self-perceived meaningful activity participation. The MAPA has been tested on 154 community-dwelling older adults in good health [11]. The results showed that it is a reliable and valid measure of meaningful activity participation, with excellent test–retest reliability (*r* = .84), good internal consistency (α = 0.85), and good convergent validity with measures of psychosocial well-being and health-related quality of life (α = 0.77–0.89; 0.62–0.93). The MAPA can predict several aspects of successful ageing, such as life satisfaction and less depressive symptoms, meaningfulness of engaged activities, life purpose, and the health-related quality of life of the individual [23].

Given the great benefits of meaningful activity participation to an individual’s ability and function, health, well-being, and quality of life, the need to use assessments of levels of meaningful activity participation in clinical populations has been proposed by health care practitioners in Taiwan [24, 25]. Accordingly, the present authors have translated the MAPA into Mandarin Chinese and culturally adapted it for people in Taiwan (i.e., the Taiwan version of the MAPA; the T-MAPA) [26]. Specifically, the activity items, instructions, and response options used in the MAPA can present differently for target populations in specific countries and cultures. Therefore, our translation and cultural adaptation aimed to achieve conceptual, item, semantic, and operational equivalence between the original and adapted versions to ensure that content validity was retained [27, 28]. We expected that between versions (MAPA and T-MAPA), the measure would have the same relationships to the underlying concept, the items would be equally relevant and acceptable, and the transference of meaning across languages would be appropriate. In addition, the format, instructions, and administration and measurement methods would have the same effects on both populations. Consequently, several activity items in the MAPA were adapted (i.e., merged, divided, or renamed) for the T-MAPA and new activity items were added (e.g., doing household chores, enjoying good food) [26].

To the present authors’ knowledge, the culturally adapted T-MAPA with adapted and newly added items has been used by health care practitioners and research teams in Taiwan. However, these users lack information on the psychometric properties of the pre-final T-MAPA (i.e., the MAPA adapted for the Taiwanese context but not yet tested for its formal psychometric properties; for detailed information, please refer to the ‘Development of the pre-final MAPA’ subsection under ‘Methods’). A valid T-MAPA could provide practitioners useful information (e.g., predictions of healthy ageing of individuals and measurements of outcomes). Although previous psychometric investigation revealed that the original MAPA can be used to address the level of well-being and health-related quality of life in older adults, several items such as playing games, medical visits, and using public transportation were reported to have less relevance to the whole scale [23]. With the inclusion of these ‘misfitting’ items and adapted and newly added items, the existing pre-final T-MAPA has not been psychometrically investigated for its utility in practice. To ensure that the strong psychometric properties of the original MAPA have been retained after adaptation, the psychometric properties of the pre-final T-MAPA must be investigated. Therefore, the purposes of this study were to (i) utilize Rasch analysis to determine the formal Taiwan version of the MAPA (the T-MAPA), and (ii) to investigate the psychometric properties of the T-MAPA, including its dimensionality, measurement invariance, item targeting, person reliability, convergent validity and test–retest reliability.

## Methods

### Procedures

The whole study was conducted in three phases (Additional file 1). The first phase involved a 6-stage qualitative process of translating and adapting the original MAPA into Mandarin (i.e., the pre-final T-MAPA) [26]. The second and third phases focused on item modifications of the pre-final T-MAPA and psychometric evaluations of the final T-MAPA in a group of healthy older adults. The study protocol was approved by the ethics committee of the Kaohsiung Medical University Hospital, and written informed consent was obtained from each participant before participation.

### Development of the pre-final T-MAPA

The process of translation and cross-cultural adaptation comprised 6 stages. In the forward, synthesis of, and back translation stages, the MAPA was first independently translated into Mandarin by two bilingual speakers. Discrepancies between the two versions were discussed until consensus between translators was reached and a synthesized version (T12) was produced. Version T12 was then back translated into English by two native English speakers who had no medical background. Once the back*-*translations were completed, two experts in occupational science compared each item between versions to identify any semantic incongruence or potential for cultural irrelevance and added descriptions for each activity item to avoid ambiguity in interpretation. In addition, selected terms and expressions were changed to take local culture practices into account. These included home making/home maintenance, driving, medical visits, taking courses, playing games, prayer/meditation, and crafts/hobbies. In addition, four new activity items deemed especially relevant to the older adults in Taiwan were added as follows: shopping/grocery shopping, caring for family members, visiting a movie theatre, and enjoying good food.

The cognitive debriefing stage was performed in two focus groups of older adults to evaluate the understandability, clarity, and cultural relevance of the items. A total of 18 participants were purposively sampled to maximize their diversity in terms of lifestyle, sex, and education. The first group, composed of participants with a more active lifestyle, consisted of 10 people (five men and five women; mean education years = 14.4, standard deviation SD = 1.58; mean age = 63.5 years, ranging from 57 to 72), and the second group, comprising those with a more static lifestyle, was composed of eight people (three men and five women; mean education years = 10, SD = 4.11; mean age = 69.75 years, ranging from 66 to 75). Furthermore, to clarify the semantic meaning and scope of the activities referred to in the questionable items raised by focus groups, the developer of the original MAPA (i.e., EAM, [23]) was consulted. As a result, the T-MAPA was further modified as follows: (i) definitions of activity items were modified or confirmed in ‘home maintenance’, ‘using personal vehicles’, ‘seeking medical assistance’, ‘crafts activities’, ‘creative activities’, ‘religious activities’, ‘prayer/meditation/ancestor worship’, ‘caring for family members’, ‘visiting a movie theatre’, and ‘helping others’; (ii) items with similar concepts were merged as ‘crafts/creative activities’, ‘other computer use’, and ‘community organization activities/volunteering’; (iii) items containing divergent concepts were divided into two, such as ‘doing household chores’ and ‘home maintenance’; (iv) the format of responses was changed to checked boxes; and (iv) the evaluation period was clarified as ‘the last three months’ [26].

In the expert review stage, a multidisciplinary consensus committee consolidated the semantic, idiomatic, conceptual, and cultural equivalences between the translated and original versions. This process produced a 29-item pilot version, in which the referential meaning of each item coupled with exemplars was established to serve as a reference for the potential respondents. Moreover, for the consistency of the concepts employed in the items and the simplicity of administration, the two subscales of the MAPA (i.e., frequency and perceived meaningfulness) were presented in the same item. These two facets of activity participation could be rated simultaneously rather than separately for each item (Additional file 2).

Finally, the pilot version was tested on eight community-dwelling older adults (mean age = 69.75 years, ranging from 66 to 75; mean education years = 10, with a wide range of 5 to 16). To improve the clarity and relevance of each item, the layout of the response options and the wordings of the item descriptions were modified. Subsequently, a pre-final 29-item T-MAPA was generated and its fitness to the Rasch model was further examined.

### Development of the formal T-MAPA and its psychometric evaluation

#### Participants

A total of 146 older adults were recruited from several local communities in southern Taiwan to participate in the field test of the T-MAPA. Inclusion criteria were age of 55 years or above and ability to read and fill out the study questionnaires. Participants were excluded if they had a significant history of neurological disease, psychiatric disorders, substance use disorders, intellectual disorder, severe visual or hearing impairments, or other serious medical conditions that had resulted in hospitalization within three months prior to study entry. Of these participants, 49 were willing to take part in the test–retest reliability study with a two-week interval, and 120 agreed to participate in the convergent validity study.

### Measures

***The pre-final T-MAPA (Taiwan version of the Meaningful Activity Participation Assessment)***. The translated and culturally adapted T-MAPA contained 29 activity items. Each item described the content of activities and measured the frequency and meaningfulness of activity participation based on personal experiences. The frequency and meaningfulness subscales were constructed to address the objective and subjective aspects of meaningful activity participation. Therefore, the two subscales were presented in the same item. The scores of the 7-category frequency and the 5-category meaningfulness subscales were multiplied for each item, and the scores of each item were summed to generate total scores. The total score of the T-MAPA represented the overall level of meaningful activity participation of an individual. Higher scores represented more meaningful activity participation.

Compared with the original version of the MAPA [29], the pre-final 29-item T-MAPA had several changes, as follows. (1) Items with similar concepts were merged to avoid redundancy and their terms were modified, such as ‘computer use for e-mail’ to ‘writing letters/cards/email’, ‘community organization activities’ and ‘volunteering activities’ to ‘community organization activities/volunteering’, and ‘crafts/hobbies’ and ‘creative activities’ to ‘crafts/creative activities’. (2) Items containing divergent concepts were divided, such as ‘home making/home maintenance’ into ‘doing household chores’ and ‘home maintenance’. (3) Items presenting culturally-specific meanings or customs in Taiwan (or Mandarin-speaking cultures) were added or adapted, including doing household chores, caring for family members, using personal vehicles, seeking medical assistance, writing letters/cards/e-mail, community organization activities and volunteering, crafts/creative activities, enjoying good food, board or educational games, shopping/grocery shopping, and visiting a movie theatre. (4) Each item was presented along with its definition coupled with exemplars. (5) The frequency and meaningfulness subscales were presented in the same item. (6) The format of responses was changed. (7) The evaluation period of the frequency subscale was clarified (Additional file 2). The pre-final 29-item T-MAPA was used for further Rasch analysis to produce the formal T-MAPA in the present study.

**Criterion measures.** Three self-report questionnaires, the Chinese version of the Life Satisfaction Z-Form Scale (LSI-Z; [30]), the Center for Epidemiologic Studies Depression Scale (CES-D; [31]), and the Short Form-36 (SF-36; [32]), were employed to examine the convergent validity of the T-MAPA by its association with other indicators of healthy ageing (i.e., psychological well-being, less depressive symptoms, and mental and physical health).

***Life Satisfaction Z-Form Scale***. The LSI-Z is used to evaluate the psychological well-being of older adults [33]. The 13 items are rated on a scale of 0 to 2 points for the individual’s degree of agreement with each statement, with higher scores indicating higher levels of life satisfaction. The inter-rater reliability (*r* = .80–0.90), internal consistency (*α* = 0.74–0.90), criterion validity (*r* = .55–0.70 with other measures of life satisfaction), and convergent–divergent validity are satisfactory [34, 35].

***The Center for Epidemiologic Studies Depression Scale***. The CES-D is a 20-item scale purported to measure the current level of depressive symptoms [36]. Participants are asked to rate how often over the past week they experienced each of 20 symptoms on a scale of 0–3, with higher scores indicating greater severity of depression. The psychometric properties have been demonstrated as adequate in community-dwelling older adults, with internal consistency (α = 0.85–0.91), inter-rater reliability (*r* = .50–0.60), and convergent validity (*r* = .39–0.74) [37–40].

***Short Form-36***. The SF-36 is a multi-item scale that evaluates health status in the past 4 weeks in 8 different domains: physical functioning, role-physical, bodily pain, general health, vitality, social functioning, role-emotional, and mental health [41]. It yields two factor-based summary scores, namely, the physical component summary (PCS) and the mental component summary (MCS) which range from 0 to 100, with higher scores representing better well-being. The internal consistencies of the MCS and PCS were found to be 0.88 and 0.93, respectively. Both composite measures have extensive empirical support for their validity [42, 43].

### Data analysis

***Rasch Analysis***. Rasch analysis was used to obtain objective, fundamental, and additive measures from responses to ordered categories [44]. The psychometric properties obtained from Rasch analysis are not affected by sample characteristics. Rasch analysis transforms ordinal scores to the logit scale and thus to an interval-level measurement. Furthermore, the Rasch model can examine whether items from a scale measure a unidimensional construct. With such measurement characteristics, the total scores summed from each item score in the Rasch-calibrated T-MAPA can be appropriate to capture the character of meaningful activity participation in older populations. In addition, the linear transformation of the raw ordinal score provides valid parametric approaches for other psychometric evaluations, such as testing convergent validity [45]. Data derived from the pre-final T-MAPA with multiple response categories were subjected to Rasch analysis using Andrich’s rating scale model in WINSTEP version 3.68 [46]. Rasch analysis was performed to evaluate the category function of rating scales, unidimensionality, differential item functioning (DIF), item difficulty and targeting, and reliability and test information function (TIF).

The category function was examined and optimized in three steps. First, the optimal number of response categories to respectively measure the frequency and perceived meaningfulness of activity participation was determined. Second, the responses (scores) derived from the two multiplied subscales were further divided into fewer categories because the multiplied total scores comprised too many categories for our sample size. Third, the appropriate number of response categories to measure the total level of meaningful activity participation was tested. Thus, a set of criteria was applied to ensure a properly functioning rating scale, including (i) at least 10 observations per category, (ii) a regular frequency distribution of responses across response categories, (iii) monotonically advancing average measures and step calibrations across categories, and (iv) outlier-sensitive (outfit) mean square (MNSQ) for each category < 2.0 [47, 48]. Disordering of categories indicates that the response category does not function adequately. Possible reasons may include difficulty discriminating between categories due to the presentation of too many response options or confusing category labels, underused categories, or a narrow interval on the latent variable that a category represents. In this case, adjacent categories were collapsed into one single response category until step calibrations were ordered [48].

Scale unidimensionality was examined using the fit statistics and was confirmed by the residuals of the data with principal component analysis (PCA). The usual measures of item fit reported in Rasch modelling are the information-weighted mean square (i.e., infit MNSQ) and the unweighted mean square (i.e., outfit MNSQ) statistics. Infit is sensitive to unexpected responses to items closer to the person ability, whereas outfit is sensitive to unexpected responses to items away from the person ability. MNSQ is the ratio of the observed variance to the Rasch predicted variance, with an ideal value of 1.0. Values of infit and outfit statistics between 0.6 and 1.4 are considered acceptable for a Likert*-*type rating scale [49]. Substantial deviance from this recommended range implies an underfit or overfit to the Rasch model, which leads to violation of the unidimensionality assumption. In this study, the content of the misfit items was evaluated before their removal from the scale. After the misfit items were removed, the Rasch analysis was rerun. This iterative process of item reduction and Rasch analysis continued until no further misfit items were observed.

Further empirical evidence in support of unidimensionality was obtained by using PCA of residuals to determine if the variance explained by the Rasch measures (i.e., the first latent dimension) exceeded 50% and the variance unexplained by the second dimension (i.e., the first contrast in the residuals) had an eigenvalue smaller than 3 and less than 5% [50].

DIF analysis was conducted to examine the items for signs of interactions with sample characteristics [51]. Specifically, the DIF in the T-MAPA was evaluated to identify whether the differences in scores were due to levels of meaningful activity participation or blended with sample characteristics. Thus, sex and education were included for DIF analysis. DIF occurs when people with the same ability level respond differently to the same item due to demographic or other background features. Items with an absolute DIF size > 0.64 logits between subgroups of specific sample characteristic were identified as having a large DIF [52] and considered for deletion. Notably, no items were removed based solely on statistical considerations; instead, misfit or DIF items were reviewed for relevance and importance to the target population before a decision was made on their possible elimination.

Item difficulty was examined to identify the level of meaningfulness and engagement frequency of each activity engaged in by the sample. Item targeting was determined by the comparison of the mean location scores of persons with the value of zero logits set for the items. An average person measure of 0 relative to the mean item difficulty implies perfect targeting of the items. In contrast, a difference in means greater than 1.0 logit suggests substantial mistargeting [53]. In addition, floor and ceiling effects surpassing 20% of the responses were regarded problems of distribution [54].

The investigation of reliability included person reliability and separation statistics, as well as test information function (TIF). A value of person separation index (PSI) of 2.0 with related person reliability of 0.80 evidences that the scale can distinguish between 3 levels of person ability (i.e., meaningful activity participation), which can be considered a minimum value for group-level measurement [55]. Furthermore, to gauge the range of abilities that can be precisely estimated by the T-MAPA, the test information curve was plotted, and TIF was calculated by the inverse of the square of the variance of the Rasch person measure (i.e., standard error, SE). An SE < 0.5 with a corresponding value of test information > 4 is considered to indicate acceptable precision of a scale [56].

***Test–Retest Reliability and measurement error***. The test–retest reliability of the Rasch-derived T-MAPA was assessed using the intra-class correlation coefficient with a two-way random effects model (ICC _2.1_). Values higher than 0.8 were regarded as substantial; 0.6–0.8 as good; 0.4–0.6 as moderate; and below 0.4 as poor concordance [57]. The amount of measurement error indexed by the standard error of measurement (SEM) indicates the precision of individual scores over repeated measures. SEM was calculated by multiplying the sample standard deviation (SD) at baseline by the square root of 1 minus the test–retest reliability coefficient [58]. SEM ≤ SD /2 was considered to have acceptable precision [59].

***Convergent Validity***. After the score distributions of criterion variables were examined, the convergent validity was assessed with the Pearson correlation coefficient by correlating the logit scores of the Rasch-derived T-MAPA with the raw scores of the LSI-Z, the CES-D, and the SF-36 respectively. Generally, Pearson’s *r* > .75 is regarded as excellent, 0.5 to 0.75 as good, 0.25 to 0.50 as moderate, and < 0.25 as low. Therefore, a correlation of *r* ≥ .5 was used to determine the convergent validity of the T-MAPA [60].

## Results

### Participant characteristics

Table 1 presents the characteristics of participants in this study. The entire sample included 146 older adults (50% male and 50% female; mean age = 65.35 years, ranging from 56 to 87; 11.6% with 0–6 years of education, 10.3% with 7–9 years, 31.5% with 10–12 years, and 46.6% with 13–16 years). The 46 participants in the test–retest reliability testing were of different ages (mean age = 63.71 years, ranging from 56 to 87), and nearly half were male (49.0%). The 120 participants in the convergent validity testing were of different ages (mean age = 64.95 years, ranging from 56 to 87) and nearly half were male (49.2%).

**Table 1:** Characteristics of study participants

Characteristic	Entire sample (*n* = 146)		Test–retest reliability Sample (*n* = 49)		Convergent validity sample (*n* = 120)	
	*n*	%	*n*	%	*n*	%
	(Mean ± SD, Range)		(Mean ± SD, Range)		(Mean ± SD, Range)	
**Age**, years	(65.35 ± 6.37, 56–87)		(63.71 ± 5.44, 56–87)		(64.95 ± 5.80, 56–87)	
55–65	83	56.8	30	61.2	68	56.7
66–87	63	43.2	19	38.8	52	43.3
**Sex**						
Male	73	50	24	49.0	59	49.2
Female	73	50	25	51.0	61	50.8
**Education**, years						
0–6	17	11.6	2	4.1	11	7.5
7–9	15	10.3	1	2.0	9	6.2
10–12	46	31.5	15	30.6	36	24.7
> 12	68	46.6	31	63.3	90	61.6
**Socio-economic status** ^**a**^						
Good	7	4.8	0	0	4	3.3
Fair	126	86.3	49	100	109	90.8
Poor	10	6.8	0	0	6	5.0
Very poor	3	2.1	0	0	1	0.8
**Marital status**						
Single	2	1.4	1	2.0	1	0.8
Married/cohabitating	113	77.4	44	89.8	97	80.8
Divorced/separated	14	9.6	1	2.0	9	7.5
Widowed	17	11.6	3	6.1	13	10.8
**Living arrangement**						
Alone	20	13.7	3	6.1	16	13.3
With others	126	86.3	46	93.9	104	86.7

*SD* Standard deviation. ^a^ Reported using subjective rating.

### Rasch analysis

Regarding the results of category functions examination on the 29-item T-MAPA, the category function of the meaningfulness rating scale was satisfactory, and that of the frequency rating scale was optimized after the management of disordered categories. For the meaningfulness rating scale, the average measures and step calibrations increased monotonically across categories for all items, and outfit MNSQ statistics were within the predetermined cut-off values for all categories (0.91–1.23; Table 2). Therefore, the 5-category rating scale for meaningfulness was retained. Regarding the frequency rating scale, the step calibrations decreased monotonically and were disordered for categories 0 to 6, implying that the probability of selecting given categories did not accord with the respondent’s level in frequency of participation (Table 3). Furthermore, categories 2 (once a month), 3 (2 to 3 times a month), and 4 (once a week) accounted for less than 10% of responses, as participants commonly selected the lowest and highest response options (Table 3). This finding indicated that the original frequency rating scale may have had too many categories such that the distinctions between them might not be meaningful to the participants (Fig. 1A). Therefore, the response categories were collapsed from 7 categories to 4: categories 1, 2, and 3, and categories 4 and 5 were respectively combined, while categories 0 and 6 remained unchanged. Analysis of the rescored data (0 = not at all, 1 = 1–3 times a month, 2 = 1–6 times a week, 3 = every day) yielded properly ordered thresholds in general (i.e., advancing step calibrations with − 0.67, 0.28, and 0.39 across categories) for all items (Table 3; Fig. 1B). Additionally, the 13 response categories (categories 0–12, indicating 0–12 points) derived from the multiplied 5-category rating (0–4 points for meaningfulness) and 4-category rating (0–3 points for frequency) were divided into 4 categories: category 0 remained 0; 1–3 was recoded as 1; 4–8 as 2; and 9–12 as 3. Analysis of the recoded 4-category data generated adequate outfit MNSQ (0.87–1.11) for each category as well as appropriately ordered thresholds (i.e., advancing step calibrations with − 0.76, 0.01, and 0.75 across categories) for all items (Table 4; Fig. 2). Thus, the recoded 4-category observations of the 29-item T-MAPA could be used for further examination of item fit statistics.

**Table 2:** Meaningfulness subscale: 5-category rating scale statistics for the T-MAPA (*n* = 146)

Category labels ^a^	Observed count (%)	Observed average	Outfit MNSQ	Step calibrations
0	252 (6)	−1.00	1.23	None
1	803 (19)	−0.45	0.91	−1.91
2	867 (20)	0.33	1.03	−0.11
3	1556 (37)	0.88	0.94	0
4	766 (18)	1.84	1.00	2.01

*T-MAPA* Taiwan version of the Meaningful Activity Participation Assessment, *MNSQ* mean square.

^a^ 0 = not at all, 1 = somewhat meaningful, 2 = moderately meaningful, 3 = very meaningful, 4 = extremely meaningful.

**Table 3:** Frequency subscale: 7-category and collapsed 4-category rating scale statistics for the T-MAPA (*n* = 146)

Category labels		Observed count (%)		Observed average		Outfit MNSQ		Step calibrations	
Before ^a^	After^b^	Before	After	Before	After	Before	After	Before	After
0	0	1165 (28)	1165 (28)	−0.41	−0.88	1.13	1.05	None	None
1	1	603 (14)	1236 (29)	−0.25	− 0.35	0.97	0.73	0.32	− 0.67
2		366 (9)		−0.17		0.75		0.27	
3		267 (6)		−0.03		0.75		0.19	
4	2	312 (7)	833 (20)	0.04	0.24	0.83	0.98	−0.16	0.28
5		521 (12)		0.23		1.11		−0.37	
6	3	1000 (24)	1000 (24)	0.59	1.00	1.31	1.28	−0.26	0.39

*T-MAPA* Taiwan version of the Meaningful Activity Participation Assessment, *MNSQ* mean square.

^a^ represents the 7 response categories of the frequency subscale before collapsing. 0 = not at all, 1 = less than once a month, 2 = once a month, 3 = 2 to 3 times a month, 4 = once a week, 5 = several times a week, 6 = every day. ^b^ represents the 4 response categories of the frequency subscale after the scale was collapsed. 0 = not at all, 1 = 1–3 times a month, 2 = 1–6 times a week, 3 = every day.

**Fig. 1A:**
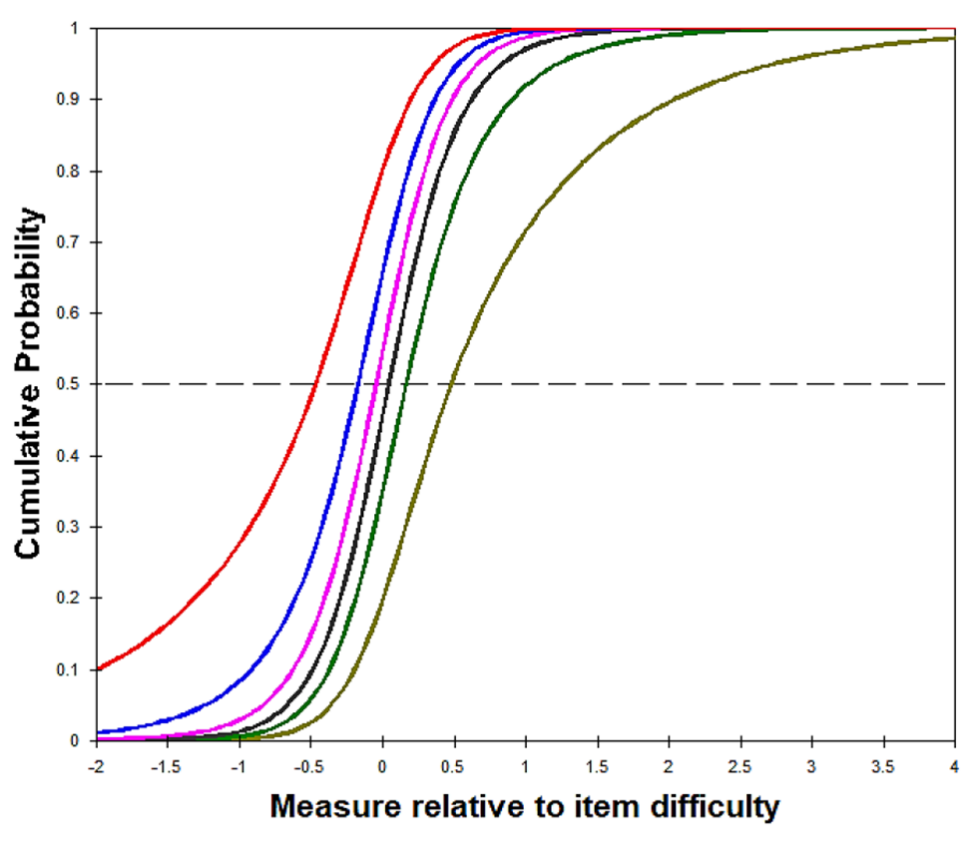
Frequency subscale: cumulative probability before collapse (exemplified with item 1) The 6 cumulative probability curves of the successive step calibrations in the original 7-category scale were too close to be distinguished, implying that discriminating between the 7 categories may be difficult for respondents

**Fig. 1B:**
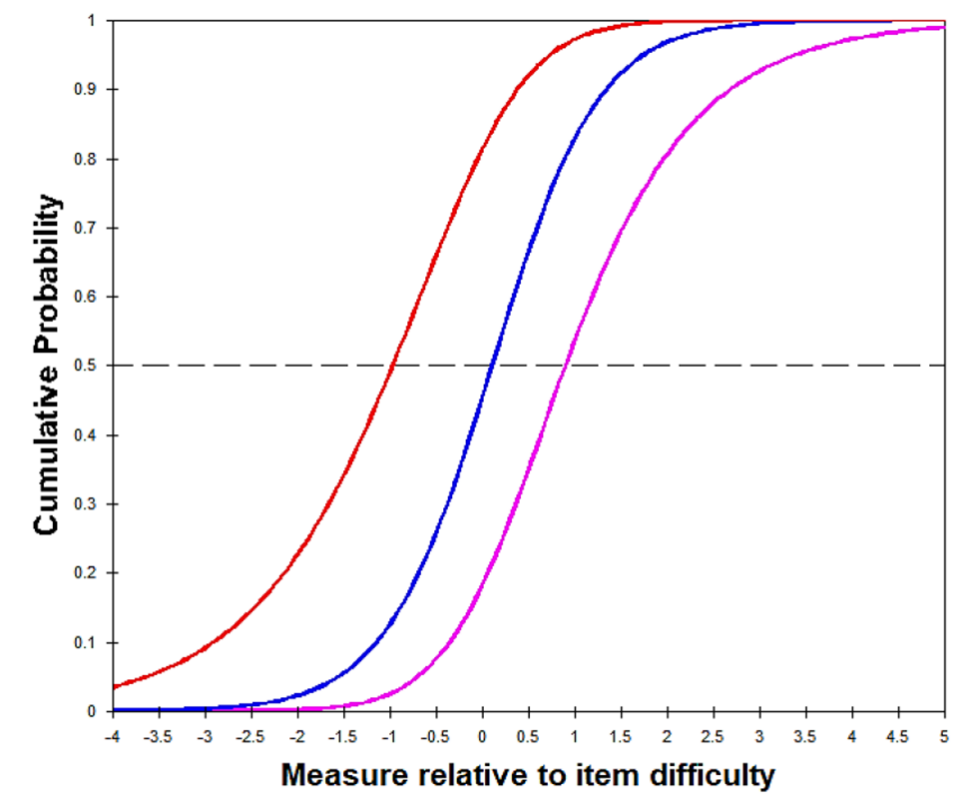
Frequency subscale: cumulative probability after collapse (exemplified with item 1) The 3 cumulative probability curves of the successive step calibrations in the collapsed 4-category scale were distinctive. Furthermore, this 4-category scale presents monotonically advancing average measures and step calibrations across categories.

**Fig. 2:**
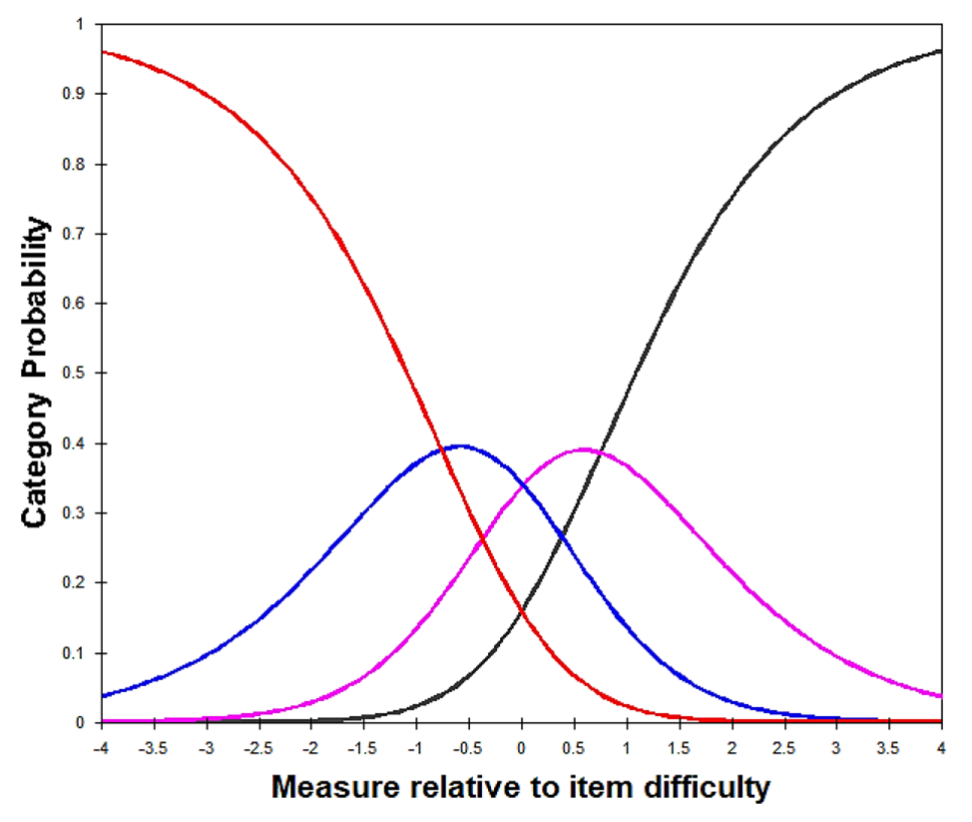
Total scale: category probability curve after collapse (exemplified with item 1) The 4-category total scale of the T-MAPA presents ordered categories and smoothly advancing step calibrations

**Table 4:** Total scale^a^: 4-category rating scale statistics for the T-MAPA (*n* = 146)

Category labels	Observed count (%)	Observed average	Outfit MNSQ	Step calibrations
0	1196 (28)	−1.08	1.02	None
1	1215 (29)	− 0.43	0.87	− 0.76
2	1030 (24)	0.18	0.92	0.01
3	793 (19)	0.80	1.11	0.75

*T-MAPA* Taiwan version of the Meaningful Activity Participation Assessment, *MNSQ* mean square.

^a^ represents the sum of the 5-category meaningfulness rating multiplied by the collapsed 4-category frequency rating.

**Unidimensionality**. First, an examination of individual item fit statistics revealed 1 misfitting item (item 4 ‘pet care activities’) and 2 borderline misfitting items (items 3 ‘caring for family members’ and 26 ‘prayer/meditation/ancestor worship’) with infit/outfit MNSQ = 2.26/2.32, 1.65/1.72, and 1.64/1.62, respectively. After we consulted two experts in occupational science and gerontology, item 4 was removed and items 3 and 26 were retained. The reduced 28-item T-MAPA was found to have good person reliability of 0.86. Second, the results of PCA on the remaining 28 items revealed that 58.1% of the variance was explained by the Rasch dimension (i.e., meaningful activity participation), and the variance unexplained by the secondary dimension had an eigenvalue of 2.3 and was 4.9%. Taken together, the overall fit and PCA results supported the conclusion that a unidimensional construct was measured by the 28-item T-MAPA.

**DIF.** The results of DIF examination indicated that none of the items manifested significant subgroup differences by sex and education, except for items 2, 6, 10, 22, and 29 (DIF size = −0.72, −1.28, 1.17, and 1.43) regarding the participants with education years below 7. The results showed that the participants with 6 or less years of education were more likely to report doing home repair/maintenance and using personal vehicles and less likely to report writing letters/cards/email and using electronic devices for other purposes, compared with those with more years of education. After consultation with experts, the five items were considered to have slight DIF with negligible real impact on person measures, but they were judged to be important and relevant to older adults in the aspects of daily living, social communication, and learning. Thus, they were retained. Subsequently, the formal T-MAPA with 28 items was produced (An example of an item in the T-MAPA is presented in Additional file 2).

**Fig. 3:**
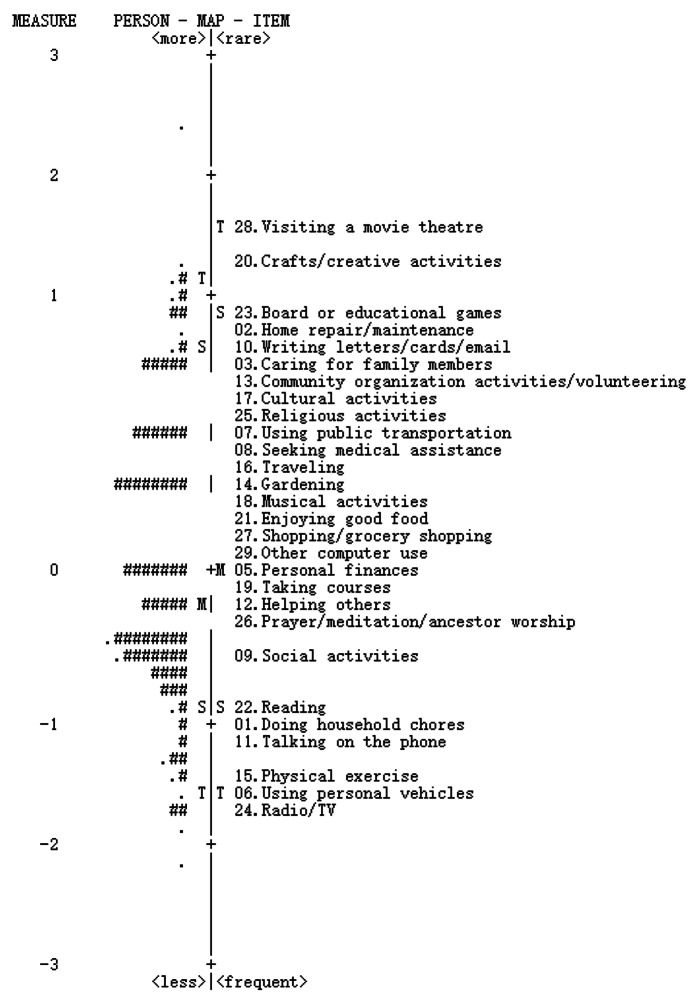
Person-item map for the 28-item Taiwan version of the MAPA (*n* = 146) Each ‘#’ is two persons and each ‘.’ is one person; M, S, and T represent the mean, and 1 and 2 standard deviations, respectively

**Item difficulty and targeting.** The item difficulties of each item of the 28-item T-MAPA are presented in Table 5. The most difficult item was item 28, which measures the activity of visiting a movie theatre (1.61 logits). The easiest was item 24, measuring listening to radio/watching TV (−1.69 logits). The person-item map presented in Fig. 3 depicts the Rasch calibrated person’s ability level (i.e., the level of activity participation) on the left side and the relative difficulty level of each item (i.e., meaningfulness and engagement frequency) on the right side. A greater level of item difficulty implies lower odds of the activity item being engaged in by participants. Mean person scores of −0.18 logits (standard deviation = 0.70) relative to the mean item difficulty indicate that the activities are in general slightly difficult for participants to engage in. Namely, the scale is targeted to more active participants who ascribe greater personal meaningfulness and engagement frequency to activity participation. A difference between the means of person scores and item difficulty of less than 1 logit suggests acceptable item targeting of the T-MAPA [53]. The floor and ceiling effects of the scale were negligible, as only 1.37% (2/146) and 0.68% (1/146) of the participants (< 20%) had scores beyond the whole range of item locations.

**Table 5:** Rasch analysis of the calibrated 28-item T-MAPA (*n* = 146)

Activity item	Measure (logits)	Model SE	Infit MNSQ	Outfit MNSQ	DIF size on sex (logits)		DIF size on education level^a^ (logits)			
					Male	Female	L1	L2	L3	L4
01.Doing household chores^b^	−0.96	0.10	1.20	1.21	0.40	−0.47	0	−0.06	−0.10	0.10
02.Home repair/maintenance	0.66	0.10	1.01	1.05	−0.31	0.32	**0.72** ^*^	0.54	0.11	0
03.Caring for family members^b^	0.48	0.10	1.70	1.76	−0.30	0.30	−0.45	0.72	−0.28	0.16
05.Personal finances	0.04	0.10	1.24	1.21	−0.06	0.05	0.54	−0.08	−0.10	−0.03
06.Using personal vehicles^b^	−1.61	0.12	1.56	1.47	−0.34	0.31	**1.28** ^*^	0.49	−0.16	0.32
07.Using public transportation	0.35	0.10	0.76	0.84	0.06	−0.06	−0.49	−0.48	0.14	0.13
08.Seeking medical assistance^b^	0.33	0.10	0.93	1.13	−0.2	0.19	−0.78	−0.37	0.12	0.18
09.Social activities	−0.46	0.10	0.74	0.71	−0.03	0.03	−0.28	−0.19	0.06	0.08
10.Writing letters/cards/email^b^	0.63	0.10	1.02	0.94	0.18	−0.16	**1.17** ^*^	0.74	0.53	−0.51
11.Talking on the phone^b^	−1.08	0.11	0.87	0.82	−0.06	0.06	−0.21	−0.04	0	0.10
12.Helping others	−0.18	0.10	0.63	0.63	−0.14	0.13	−0.34	−0.30	−0.03	0.17
13.Community organization activities/volunteering	0.40	0.10	1.16	1.13	−0.23	0.22	0	−0.44	−0.17	0.20
14.Gardening	0.12	0.10	1.24	1.22	0.13	−0.12	0.69	0.12	0	−0.17
15.Physical exercise	−1.44	0.12	1.07	1.00	−0.11	0.12	0.08	−0.11	−0.10	0.09
16.Traveling	0.22	0.10	0.46	0.48	0.03	−0.02	−0.2	0	−0.08	0.09
17.Cultural activities^b^	0.46	0.10	0.54	0.54	0.04	−0.04	0.13	0.09	0	−0.04
18.Musical activities	0.09	0.10	0.93	0.95	−0.10	0.09	0.29	0.05	−0.07	−0.03
19.Taking courses^b^	0.01	0.10	0.86	0.92	0.50	−0.44	0.37	0.33	0.04	−0.17
20.Crafts/creative activities	1.29	0.12	1.10	1.20	0.19	−0.16	−0.21	−0.51	0.20	0.03
21.Enjoying good food^b^	0.12	0.10	0.74	0.76	0	0	0	0.12	−0.13	0.06
22.Reading^b^	−0.79	0.10	1.03	0.97	0.10	−0.10	**1.43** ^*^	0.66	0.09	−0.80
23.Board or educational games^b^	0.9	0.11	1.08	0.98	0.11	−0.10	0.70	0.67	0.07	−0.22
24.Radio/TV	−1.69	0.12	1.02	0.99	−0.17	0.17	−0.04	0	0	0
25.Religious activities	0.48	0.10	1.14	1.18	0.11	−0.09	−0.45	−0.61	−0.09	0.29
26.Prayer/meditation/ancestor worship^b^	−0.13	0.10	1.69	1.67	−0.13	0.12	−0.46	−0.89	0.07	0.26
27.Shopping/grocery shopping^b^	0.08	0.10	0.60	0.63	−0.04	0.04	−0.52	−0.47	0.04	0.20
28.Visiting a movie theatre^b^	1.61	0.14	0.77	0.70	0.28	−0.22	0.55	0.21	−0.24	0.03
29.Other computer use^b^	0.08	0.10	1.33	1.29	0.15	−0.13	**2.00** ^*^	0.82	0.12	−0.56

*T-MAPA* Taiwan version of the Meaningful Activity Participation Assessment, *SE* standard error, *MNSQ* mean square, *DIF* differential item functioning.

^*^ indicates the item with DIF effect size > 0.64 logits. ^a^Education years are divided into 4 levels: L1 = 0–6, L2 = 7–9, L3 = 10–12, and L4 > 12 years. ^b^ indicates culturally adapted or newly added activities.

**Fig. 4:**
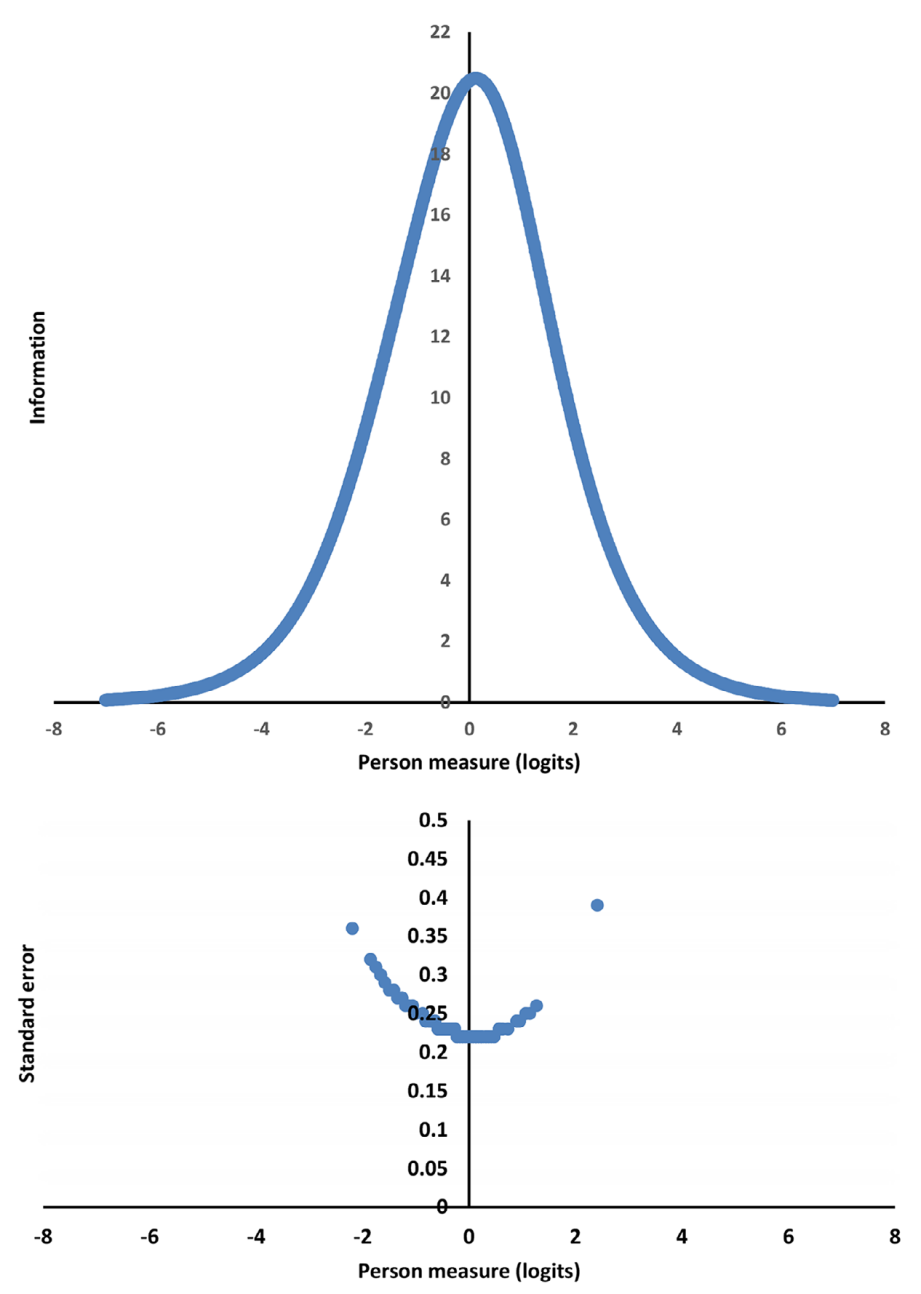
Curve of the test information and the standard error of person measure The upper panel presents a person measure with − 0.22–0.47 logits, offering maximum information (20.66) of the meaningful activity participation continuum; the lower panel presents the small standard error (0.22–0.39) of person measures (−2.19–2.41 logits), estimated for the 28-item T-MAPA from 146 participants

**Person reliability and separation, and TIF.** The person reliability coefficient of 0.86 with a PSI of 2.51 indicate that 3 levels of meaningful activity participation in this sample could be discriminated by the 28-item T-MAPA with satisfactory reliability. The test information curve and the SE regarding person measure are shown in Fig. 4. The test information curve was bell-shaped, with its maximal information point (20.66) on the person measure of −0.22–0.47 logits in the middle of the perceived meaningful activity participation continuum (i.e., person measure). A small SE (0.22–0.39; < 0.5) with corresponding large test information (6.57–20.66; > 4) occurred in the whole range of person measures (between − 2.19 and 2.41 logits), indicating the T-MAPA could capture the whole range of meaningful activity participation of the sample. Overall, the T-MAPA provided precise estimates of various levels of meaningful activity participation in this sample.

The upper panel presents a person measure with − 0.22–0.47 logits, offering maximum information (20.66) of the meaningful activity participation continuum; the lower panel presents the small standard error (0.22–0.39) of person measures (−2.19–2.41 logits), estimated for the 28-item T-MAPA from 146 participants.

### Test–retest reliability and measurement error

The test–retest reliability was excellent, with high correlations (ICC_2,1_ = 0.94, *p* < .0001). As an index of absolute reliability, SEM was estimated as 10.62 (< 21.68 with SD/2), indicating that the T-MAPA had acceptable precision for individual repeated measures.

### Convergent validity

Under the premise of a nearly normal distribution of all criterion measures (skewness/kurtosis = −1.14/0.81, 0.61/−0.58, −0.26/−0.79, and − 0.32/−1.07 for the LSI-Z, CES-D, SF-36 physical, and SF-36 mental health, respectively), the investigated convergent validity was satisfactory. The T-MAPA was moderately correlated with the LSI-Z assessing life satisfaction (*r* = .49, *p* < .01) and highly correlated with the CES-D (*r* = −.54, *p* < .01) and the SF-36 physical (*r* = .53, *p* < .01) and mental health (*r* = .50, *p* < .01) scales assessing depressive symptoms and health, respectively.

## Discussion

This study translated and cross-culturally adapted the original MAPA for use with older adults in Taiwan, utilized Rasch analysis to generate a 28-item T-MAPA, and investigated its psychometric properties. Our study process led us to make several specific choices to ensure that the T-MAPA would be relevant to older adults in Taiwan. With a rigorous 6-stage process of adaptation, a conceptually, semantically, and operationally equivalent and culturally relevant 29-item T-MAPA was produced. Specifically, the cross-culturally adapted T-MAPA has two merits. First, several activity items were added or modified to reflect the cultural preference or contexts of activity participation for Taiwanese older adults. These items were found to contain generally appropriate levels of activity participation and goodness of fit (item measure = −1.61–1.61 logits; infit and outfit MNSQ = 0.54–1.76).

For example, ‘home making/home maintenance’ in the original MAPA was divided into two items and their expressions were changed to ‘doing household chores’ and ‘home repair/maintenance’ because older adults in Taiwan considered ‘home making’ and ‘home maintenance’ as two different activities in terms of content, meaningfulness and frequency of engagement. In addition, changing the expression of the original ‘home making’ to ‘doing household chores’ was considered more easily understandable. ‘Driving’ was modified to ‘using personal vehicles’, which included both driving by oneself and taking taxis or riding in family/friends’ vehicles, since they are commonly used in Taiwan. ‘Medical visits’ was modified to ‘seeking medical assistance’, including rehabilitation, physical or psychological consultation, or Chinese medicine, to present the diversity of effective medical services supported by the inexpensive national health insurance system in Taiwan. ‘Community organization activities’ and ‘volunteer activities’ were combined because they were contained by each other in most situations, and the activities of enjoying good food, board or educational games, and shopping/grocery shopping were included to reflect leisure activities supported by the dense and inexpensive physical contexts of leisure facilities and shops in Taiwan. With generally acceptable fit to the Rasch model, these culturally meaningful items across the recreational, social, mental, and physical dimensions may extend the ability of assessing the participation in various activities of older adults in Mandarin-speaking cultures.

Second, each item was constructed to be easily understandable to individuals by dividing or merging activity items, designating a referential definition and exemplars for each item, and including meaningfulness and frequency subscales in the same item. The merit of these features was supported by the high person reliability (0.86) and the ability of precise estimation of various levels of meaningful activity participation in older adults (small SE of 0.22–0.39 with large test information of 6.57–20.66).

After the Rasch analysis, the 7-category rating subscale for frequency required modification because of the small number of persons responding to categories 1–5 (the frequencies in a month and in a week). The application of the collapsed 4-category frequency and the 5-category meaningfulness ratings for the total T-MAPA resulted in ordered categories and smoothly advancing thresholds (outfit MNSQ = 0.87–1.11; step calibrations = −0.76, 0.01, 0.75), indicating that the response categories of the subscales in each item were appropriate for the utility of the resultant measures. The results demonstrated that our modification solved the problems addressed by older adults in focus groups who had difficulty categorizing personal activity participation between the many different frequencies in the original version.

After the removal of one misfitting item (i.e., pet care activities), the 28-item T-MAPA generally presented a unidimensional construct with satisfactory PCA results, indicating that the 28 items represent a common latent variable which can be used for assessing the overall meaningful activity participation of individuals. The decision to remove the misfitting ‘pet care activities’ and retain the items with borderline fit statistics (i.e., ‘caring for family members’ and ‘prayer/meditation/ancestor worship’) are further discussed.

‘Pet care activities’ in the original MAPA may reflect divergent meaningfulness and frequency of participation in Taiwanese culture. For people in western countries, participating in ‘pet care activities’ may have positive benefits of preventing older adults from social isolation, contributing to better physical and psychological well-being, and being personally meaningful [61, 62]. However, divergent occupational meanings ascribed to ‘pet care activities’ were found in older adults in Taiwan. In the cognitive debriefing, some of the participants who reported high overall meaningful activity participation in the T-MAPA considered that ‘pet care activities’ was a compulsory ‘job’. Thus, most of the participants responded that the pet care activities were ‘not at all meaningful’ (35.6%) or ‘somewhat meaningful’ (29.5%). The inconsistent responses of the participants to ‘pet care activities’ and the other activities may have caused the misfitting of this item (infit/outfit MNSQ = 2.26/2.32), and thus it was deleted to ensure that the overall scale measured the same construct.

On the other hand, the item ‘caring for family members’, with borderline fit statistics, was found to be very to extremely meaningful for older adults (72.6%), indicating that this item was significant for this population. The presentation of its borderline value of MNSQ (1.65–1.72) can be explained by noting that over half of the sample was below 65 years old (56.8%) and lived with healthy family members without the need for special care (45.9% reported zero frequency of participation). With the consideration of the limited representation of our sample for the whole population and the great significance of ‘caring for family’ for older adults, item 3 was retained to support the content validity of the T-MAPA. However, further testing of this item on another sample with more ‘older’ adults (above 65 years old) is warranted to confirm its fit to the Rasch model.

The item ‘prayer/meditation/ancestor worship’, defined as ‘spiritual activities without relevance to religious belief’, demonstrated some meaningfulness with low frequency of participation (40.4% less than once a month). This can be explained by Taiwanese customs, in which people are more likely to perform religious rituals (item 25 ‘religious activities’ such as chanting scriptures and attending churches for worship) to engage in spiritual related activities and less likely to participate in personal spiritual activities (item 26 ‘Prayer/meditation/ancestor worship’). To test this hypothesis, items 25 and 26 were temporarily merged into one activity item, given that the concept of ‘spirituality’ can contain that of ‘religion’ [63]. This combination revealed a satisfactory item fit (infit/outfit MNSQ = 1.20/1.21). Further testing of item statistics on the combination of items 25 and 26 in a larger sample is recommended to confirm the adequacy of the two-item combination (religious and spiritual activities) in representing meaningful activity participation.

Although items 2, 6, 10, 22, and 29 exhibited slight DIF, they are important and relevant to older adults’ meaningful activity participation, as they include the aspects of instrumental activities of daily living, social communication, and learning. Whether such bias has substantive implications in person measures was further investigated. Linacre [47] suggested two strategies for evaluating the real influence of DIF and determining when to adjust for it. First, the DIF impact on person measures can be affected by the length of the test. The adjustment of the criteria of DIF significance by item numbers is practical and has been used previously in another scale validation study [20]. Accordingly, the absolute DIF value of 0.72–2.00 logits across the education subgroups on items 2, 6, 10, 22, and 29 may have real average impacts of 0.03–0.07 logit bias on a person’s meaningful activity participation in the 28-item T-MAPA. With such small bias, the real impact of DIF on person measures is negligible. Second, to determine when to adjust for DIF, whether the DIF is replicable should be tested when the subgroup is split in a different way. Given the far smaller numbers of persons in education levels 1 and 2 (11.6% and 10.3% respectively) than in other subgroups, the two subgroups were combined into one, and the DIF of each item across the three education subgroups (i.e., < 10, 10–12, and > 12 years) was analyzed. The results showed no significant DIF size across education subgroups for each item (absolute DIF size = 0–0.51). Therefore, the DIF bias found in the previous analysis may have been just an accident of sampling, as it disappeared after regrouping of the sample. Finally, the five items with slight DIF in the first place were retained because the DIF no longer existed after further investigations.

In the inspection of the hierarchy of activity items, several activity items with approximate logit values were found to possess different occupational forms, functions, and contents of meaningfulness. Such item combination can be beneficial in practice. Specifically, each of the activity items with approximate levels of meaningful activity participation in the scale can be used as a complementary occupation in occupational adaptation when an individual’s previous occupation has to be discontinued due to specific disabilities in the ageing process.

Regarding item difficulty, listening to radio/watching TV, using personal vehicles, and doing physical exercise were considered the three most meaningful items of activity participation (item measures = −1.69, −1.61, and −1.44 logits respectively) in Taiwan. Most of the sample reported that they listened to the radio or watched TV every day and felt it moderately to extremely meaningful. This result is consistent with another finding that older adults considered watching TV an important activity in daily living, as it helped them obtain information about what was happening in society and the world, provided entertainment, and produced emotional bonding [64]. On the other hand, doing physical exercise was also substantively significant and highly participated in by this population for health reasons [65, 66]. With increasing knowledge about the benefits of physical exercise to physical and mental health, physical exercise has become one of the most popular daily routines for older adults in Taiwan. Another activity, ‘using personal vehicles’, was reported as highly meaningful and frequently engaged in by Taiwanese older adults for several reasons. Using personal vehicles helps individuals to access other activities for physical and mental needs, such as seeking medical assistance, doing physical exercise, enjoying good food, and grocery shopping. It also provides an opportunity for individuals to explore environments for spiritual and social needs, such as travelling and attending cultural activities [67]. In addition, the unique geographical and environmental features of Taiwan, a small island with short distances between cities, and the lack of dense public transportation networks made older adults prefer to use personal vehicles such as scooters and cars for their transportation [67].

In contrast, visiting a movie theatre and doing crafts/creative activities were considered less meaningful and less engaged in (item measures = 1.61 and 1.29 logits respectively) for Taiwanese older adults. Older adults may visit movie theatres less frequently because the environment is not friendly to them due to the stairs leading to the seats and low lighting, and also that the movies on the market may not be in harmony with the preferences of older adults. Thus, the frequency of visiting movie theatres was lower than those of other activities in the T-MAPA, although watching films was found to be meaningful to this population. To substitute for their preference of visiting movie theatres in their younger days, some of the older adults in focus groups chose to stay home to watch films (included in ‘radio/TV’). Analysis of the ‘crafts/creative activities’ in the T-MAPA revealed that the ascribed meaningfulness varied, ranging from ‘somewhat to very meaningful’ for older adults; however, this activity was ‘never participated in’ by over half of the sample (59.6%). A possible explanation is that doing crafts/creative activities requires individuals to employ sharp vision, good fine motor and bilateral coordination, and more fluid intelligence, all of which are abilities that decline in older adults [68, 69]. Therefore, engaging in crafts/creative activities likely became challenging in this population, and the challenges were a deterrent to participation.

Furthermore, the differences between occupations listed in the T-MAPA and the original MAPA reflect culturally specific meanings as well as the temporal and contextual characteristics of occupations. The overall list of meaningful activities in the Taiwan version contains diverse activity items representative of specific subcultures of different generations. For example, the activity items in the T-MAPA range from traditional to contemporary (e.g., writing letters/cards to e-mail/e-cards, visiting a movie theatre to watching TV/films, reading books to e-books, doing household chores/cooking to enjoying good food). Specifically, the diversity of occupations listed for older adults reflects the evolution of the population aged 55–87 years in responding to the dramatic cultural and environmental changes in this era. Such diverse occupations provided the treasures inherent in human culture, allowing older adults nowadays to adapt to life challenges and produce positive health-related outcomes rapidly and effectively.

This study has limitations. The sample was composed of healthy older adults in Taiwan, so the generalizability of our psychometric findings to populations with other characteristics may be limited. For instance, the form, function, and meaning of item contents, and the overall meaning and frequency of activity participation, may be different for persons who have health conditions such as stroke or schizophrenia or those who belong to a different age group. In this regard, we recommend that the items in the T-MAPA be inspected for possible modification of the item contents, followed by psychometric evaluations, before the T-MAPA is applied to other populations. To extend the clinical utility of the scale, future studies are warranted to examine the optimal cut-off points of function/dysfunction in people with specific health conditions. Specifically, no information on the levels of meaningful activity participation among the older population has been established in the present study. Therefore, one cannot conclude that the lower scores of such individuals obtained in the assessment of the T-MAPA indicate substantially insufficient meaningful activity participation, and the precision of planning interventions can be negotiated. For example, scores distinguishing the levels of meaningful activity participation between older adults with and without depression should be established to determine the level of dysfunction for intervention planning in people with depression.

This 28-item T-MAPA has several important clinical implications. First, it requires only 10 to 20 min for administration, which allows older adults with poor tolerance or short attention spans to complete the whole scale without much difficulty. This field-tested and adapted scale is client-centred and can be applied with verbal or written instructions. It is also administrator-friendly with the establishment of a standardized administration manual. Moreover, this scale is suitable for older adults in Taiwan of any education level, as the study sample included individuals with elementary school-level education or below. With the inclusion of new culturally relevant items presenting small SE with large TIF, the T-MAPA provides precise estimates of various levels of meaningful activity participation in this sample. The use of the Rasch transformed score allows the application of more powerful parametric statistics to calculate and compare the assessed results. Another prominent benefit of this Rasch-calibrated T-MAPA is the construction of relative ordering of each activity item. It provides a reference for gradual interventions and the occupational adaptation process. For example, by initially introducing items reflecting lower levels of meaningful activity participation (e.g., physical exercise, using personal vehicles, and radio/TV), individuals with major difficulty in overall meaningful activity participation may benefit by acquiring adaptive skills for activity participation.

In conclusion, the 28-item T- MAPA introduces several innovations to the original version that make this assessment more suitable for the population in Taiwan (or those affiliated with Greater Chinese cultures). After a rigorous 6-stage cross-cultural adaptation process, the 28-item T-MAPA had good psychometric properties, including effective category function, unidimensionality, measurement invariance, acceptable item targeting, good person reliability, estimation precision, and test–retest reliability (with negligible measurement error), as well as confirmed convergent validity with psychological well-being and health. The T-MAPA may be applicable as a valid assessment for measuring meaningful activity participation in older adults, planning interventions, measuring outcomes, and predicting healthy ageing.

## Electronic supplementary material

Below is the link to the electronic supplementary material.

Supplementary Material 1. Figure S1. 1 Study procedures

Supplementary Material 2. Table S1 An example of an item of the 28-item T-MAPA in English

## Data Availability

The datasets used and/or analysed during the current study are available from the corresponding author on reasonable request.

## Abbreviations

MAPA: Meaningful Activity Participation Assessment
T-MAPA: Taiwan version of the Meaningful Activity Participation Assessment
WHO: the World Health Organization
LSI-Z: the Life Satisfaction Z-Form Scale
CES-D: the Center for Epidemiologic Studies Depression Scale
SF-36: the Short Form-36
DIF: Differential item functioning
TIF: Test information function
PCA: Principal components analysis
MNSQ: Mean square
PSI: Person separation index
SE: Standard error
ICC: Intra-class correlation coefficient
SEM: Standard error of measurement
SD: Standard deviation
