# Supplementary material for: Developing and validating the Taiwan version of the meaningful activity participation assessment (T-MAPA) with Rasch analysis
Source: BMC Geriatr. 2023 Mar 22;23:159. doi: 10.1186/s12877-023-03839-9 (PMC10032021; doi:10.1186/s12877-023-03839-9)
Supplement: Supplementary file 1 — Supplementary Material 1. Figure S1. 1 Study procedures [file 12877_2023_3839_MOESM1_ESM.docx]

**Additional file 1**

Phase I: Translation and cross-cultural adaptation

**Transit-stage: Expert consultation (E=1)**

**Stage II:**

**Synthesis of translation**

**(T=2)**

**Stage III:**

**Back**

**translation**

**(T=2)**

**Stage I:**

**Forward translation**

**(T=2)**

Phase III: Convergent validity & test–retest reliability tests

Phase II: Unidimensionality & reliability tests by Rasch analysis

**Figure S1. 1** Study procedures. T-MAPA = Taiwan version of the Meaningful Activity Participation Assessment, T = translator, E = expert, D = original developer, Final T-MAPA = Formal or Rasch-derived T-MAPA.

2 weeks

**Convergent validity (*n*=120):**

- **The Life Satisfaction Index-Z**
- **The Center for Epidemiologic Studies Depression Scale**
- **SF-36 Health Survey**

**Test–retest reliability (*n*=49):**

**Formal T-MAPA**

**Unidimensionality & item modifications (*n*=146)**

**(Category function, item fit, differential item functioning)**

**Pre-final T-MAPA**

**Stage VI:**

**Pilot testing**

**(*n*=8)**

**Stage V:**

**Expert review (E=4)**

**Transit-stage: Developer consultation (D=1)**

**Stage IV:**

**Cognitive debriefing (*n*=18)**

**Final T-MAPA**

**Reliability (*n*=146)**

**(Item targeting, person reliability & separation, test information function)**
