# Supplementary material for: Developing and validating the Taiwan version of the meaningful activity participation assessment (T-MAPA) with Rasch analysis
Source: BMC Geriatr. 2023 Mar 22;23:159. doi: 10.1186/s12877-023-03839-9 (PMC10032021; doi:10.1186/s12877-023-03839-9)
Supplement: Supplementary file 2 — Supplementary Material 2. Table S1 An example of an item of the 28-item T-MAPA in English [file 12877_2023_3839_MOESM2_ESM.docx]

**Additional file 2**

**Table S1** An example of an item of the 28-item T-MAPA in English

| 1. **Doing household chores, e.g., cleaning, doing the laundry and dishes, taking care of children, paying bills.** | |
| --- | --- |
| Please indicate the amount of time that you spent on this activity during the last 3 months. Check the box for the one which describes your condition. Choose only one (Check with V). | Please rate according to how meaningful this activity to you; that is, how much it matters or is personally fulfilling for you. |
| □ 1. Not at all  □ 2. 1–3 times a month  □ 3. 1–6 times a week  □ 4. Every day | □ 1. Not at all meaningful  □ 2. Somewhat meaningful  □ 3. Moderately meaningful  □ 4. Very meaningful  □ 5. Extremely meaningful |
